# Selective amygdala subregional atrophy in Parkinson’s disease: from subjective cognitive decline to mild cognitive impairment

**DOI:** 10.3389/fnagi.2026.1807979

**Published:** 2026-06-16

**Authors:** Liujia Lu, Binfen Xu, Yi Ji, Ruixuan Zhang, Wanyu Hao, Yao Lu, Feng Wang, Kaidong Chen, Li Zhang, Xiangming Fang

**Affiliations:** 1 Department of Radiology, The Affiliated Wuxi People's Hospital of Nanjing Medical University, Wuxi, Jiangsu, China; 2 Department of Neurology, The Affiliated Wuxi People's Hospital of Nanjing Medical University, Wuxi, Jiangsu, China

**Keywords:** amygdala subregions, biomarker, magnetic resonance imaging, mild cognitive impairment, Parkinson’s disease, subjective cognitive decline

## Abstract

**Background:**

Cognitive impairment is a common and disabling non-motor feature of Parkinson’s disease (PD). Subjective cognitive decline (SCD) has been proposed as an early clinical stage of cognitive dysfunction in PD; however, its neuroanatomical correlates remain poorly defined. The amygdala is an early target of PD-related pathology, yet subregional alterations across different cognitive stages of PD have not been systematically examined.

**Objective:**

To characterize amygdala subregional atrophy patterns across the cognitive continuum of PD, specifically distinguishing between subjective cognitive decline and mild cognitive impairment (MCI).

**Methods:**

High-resolution T1-weighted MRI was acquired in 68 PD patients classified as cognitively normal (PD-NC, *n* = 21), subjective cognitive decline (PD-SCD, *n* = 20), or mild cognitive impairment (PD-MCI, *n* = 27), and in 27 healthy controls. Amygdala subregional volumes were quantified using automated segmentation. Group differences were assessed using analysis of variance with correction for multiple comparisons. Associations between amygdala subregional volumes and cognitive performance were examined using correlation and multinomial logistic regression analyses.

**Results:**

Compared to controls and cognitively normal PD patients, the PD-MCI group exhibited significant atrophy specifically in the medial and cortical nuclei of the amygdala. Volumes of the medial and cortical nuclei were positively correlated with global cognitive performance and visuospatial/executive function. Multinomial logistic regression identified larger medial amygdala volume as an independent correlate of cognitive preservation in PD.

**Conclusion:**

Cognitive impairment in PD is associated with selective atrophy of specific amygdala subregions. In contrast, PD-SCD involves no detectable amygdala atrophy, suggesting it represents a structurally preserved stage preceding overt neurodegeneration. These findings suggest that PD-SCD represents a structurally preserved stage and highlight amygdala subregional volumetry as a potential biomarker for cognitive impairment in PD.

## 1 Introduction

Parkinson’s disease (PD) is a common and progressive neurodegenerative disorder, which is characterized not only by cardinal motor features but also by a broad spectrum of non-motor symptoms that substantially affect functional independence and quality of life (Leite Silva et al., 2022; Schapira et al., 2017; Svenningsson et al., 2012); a key neuropathological feature distinguishing this disorder lies in the depletion of dopaminergic neurons in the substantia nigra pars compacta and the formation of Lewy bodies resulting from *α*-synuclein accumulation (Braak et al., 1998). Cognitive impairment is among the most disabling non-motor complications of PD and represents a major determinant of long-term prognosis, caregiver burden, institutionalization, and mortality. Cognitive impairment in people with PD imposes a substantial societal burden: PD affects over 1% of the population aged 65 years and older, and 24–31% of individuals with this condition develop dementia and another 26% present with mild cognitive impairment (Oikonomou et al., 2025). Given the increasing prevalence of PD amid an aging population, the challenge of PD-associated cognitive impairment is likely to intensify. Identifying neural substrates associated with early cognitive vulnerability is therefore of considerable clinical importance.

Cognitive dysfunction in PD is increasingly recognized as a continuum, extending from normal cognition (PD-NC), through mild cognitive impairment (PD-MCI), to Parkinson’s disease dementia. In addition to objectively defined cognitive impairment, subjective cognitive decline (SCD)—defined as self-perceived cognitive worsening in the absence of measurable deficits on standardized neuropsychological testing—has now been widely applied in the prevention, diagnosis and management of Alzheimer’s disease (AD) (Wen et al., 2021; Kiene et al., 2025). SCD is recognized as a potential early stage of cognitive dysfunction and also one of the core screening tools for identifying the prodromal phase of this disease (Oedekoven et al., 2021). Emerging evidence suggests that SCD is also clinically meaningful in PD, as patients with PD-SCD are at increased risk of future cognitive decline and progression to PD-MCI (Loo et al., 2025; Flores-Torres et al., 2023; Weintraub et al., 2024). However, the structural brain correlates underlying PD-SCD and its transition to PD-MCI remain poorly understood (Chen et al., 2026).

Neuroimaging studies in PD have predominantly focused on cortical atrophy and hippocampal involvement as substrates of cognitive impairment (Lee et al., 2011; Zhu et al., 2022; Silva-Rodríguez et al., 2025; Sivaranjini and Sujatha, 2021), whereas subcortical limbic structures have been comparatively underexplored. We selected the amygdala as the research object based on the following theoretical foundations. Pathologically, from the perspective of pathological progression, Braak staging of PD neuropathology indicates that *α*-synuclein deposition occurs in a spatiotemporal sequence, and the amygdala is one of the earliest subcortical regions to be involved, even in non-demented PD patients (Flores-Cuadrado et al., 2014; Lai et al., 2025; Van Den Berge et al., 2021). Furthermore, data-driven typing and staging of brain volumetric changes in PD based on cross-sectional imaging have demonstrated that the amygdala is consistently the earliest subcortical region to exhibit atrophy, irrespective of PD subtypes (Park et al., 2025). These dual characteristics of early pathological involvement and structural atrophy suggest that the amygdala can serve as a sensitive structural biomarker for tracking early cognitive changes in Parkinson’s disease. Structurally, in terms of functional relevance, the amygdala is a key limbic structure that integrates multiple functions such as emotion regulation, motivation, learning, and higher-order cognitive processing. These functions are closely related to the cognitive and neuropsychiatric manifestations of PD (Ay et al., 2022; Seo et al., 2025), such as executive dysfunction and emotional disturbances, which are prominent features of early cognitive decline in PD. Functionally, considering subregional specificity, the amygdala is composed of multiple anatomically and functionally distinct subnuclei, including the medial, cortical, lateral, basal nuclei and so on. Different subnuclei have distinct neural connections, for example, the medial nucleus is closely connected to the prefrontal cortex that regulates executive function, and the cortical nucleus is involved in sensory-cognitive information integration. This structural and functional heterogeneity implies that different amygdala subregions may exhibit selective vulnerability during the progression of PD cognitive impairment (Torres et al., 2021). Finally, from the perspective of research gaps, existing studies have mostly focused on the overall volume changes of the amygdala in PD, while systematic investigations on the subregional volumetric alterations across different cognitive stages (especially the PD-SCD stage) are still lacking.

Recent advances in MRI-based segmentation techniques now permit *in vivo* quantification of amygdala subregional volumes (Ben-Zion et al., 2022; Leaver et al., 2020; Cheng et al., 2024). Several studies have linked atrophy of specific amygdala subnuclei to cognitive impairment in PD (Oh et al., 2024; Oltra et al., 2022); however, whether such subregional alterations are present at the stage of subjective cognitive decline has not been systematically investigated. Clarifying this issue is clinically relevant, as it may help distinguish patients with benign subjective complaints from those with emerging neurodegenerative changes.

Given the clinical focus on disease staging and translational relevance, the present study aimed to characterize amygdala subregional volumetric changes across clinically defined cognitive stages of PD. We hypothesized that selective amygdala subregional atrophy would be evident in PD-MCI, while PD-SCD would show relative structural preservation. To test this hypothesis, we compared amygdala subregional volumes among PD-NC, PD-SCD, and PD-MCI patients and healthy controls, and examined their associations with cognitive performance. Our goal was to identify clinically meaningful structural markers associated with cognitive impairment in PD.

## 2 Methods

### 2.1 Participants

Seventy patients with PD were prospectively recruited from the Parkinson’s Disease Clinic of the Affiliated Wuxi People’s Hospital of Nanjing Medical University between August 2021 and May 2024. Twenty-seven healthy controls (HCs) matched for age, sex, and education were also enrolled. The study was approved by the local Ethics Committee (KY21133), and written informed consent was obtained from all participants in accordance with the Declaration of Helsinki.

PD diagnosis was established according to the Movement Disorder Society (MDS) clinical diagnostic criteria (Postuma et al., 2015). Inclusion criteria were: age ≥ 40 years, right-handedness, and ability to complete clinical and neuropsychological assessments. Exclusion criteria included other neurological disorders (e.g., epilepsy or atypical parkinsonian syndromes), history of significant brain injury or neurosurgery, severe sensory or language impairments, and serious systemic medical illnesses.

### 2.2 Clinical and neuropsychological assessment

Demographic variables (age, sex, education) were recorded for all participants. For PD patients, disease duration, levodopa equivalent daily dose (LEDD), Unified Parkinson’s Disease Rating Scale (UPDRS) scores, and Hoehn and Yahr stage were obtained. Depressive and anxiety symptoms were assessed using the Hamilton Depression Rating Scale (HAMD) and Hamilton Anxiety Rating Scale (HAMA), respectively. Executive function was evaluated using the Frontal Assessment Battery (FAB).

Global cognitive function was assessed using the Montreal Cognitive Assessment (MoCA), with subjective cognitive complaints initially identified via UPDRS I Item 1. To assess the presence of cognitive complaints we used the Non-Motor Symptoms Scale (NMSS) Domain 5, which includes three rater-administered items: item 16 “Does the patient have problems sustaining concentration during activities?”, item 17 “Does the patient forget things that he/she has been told a short time ago or events that happened in the last few days?” and item 18 “Does the patient forget to do things?”. Based on the combined results of these assessments, PD patients were stratified into three cognitive subgroups: PD-NC (PD with normal cognition) (MoCA score ≥26, no subjective cognitive decline), PD-SCD (PD with subjective cognitive decline) (MoCA score ≥26, UPDRS I Item 1 score ≥ 1, and NMSS Domain 5 score ≥ 1), and PD-MCI (PD with mild cognitive impairment) (MoCA score <26, with subjective cognitive decline), which corresponds to a Level I (screening-based) classification per the MDS Task Force criteria. It should be noted that relying on this screening-based approach, rather than a comprehensive neuropsychological battery (Level II classification), may limit the generalizability of our findings and should be considered when making comparisons with studies utilizing Level II criteria. Patients with UPDRS I Item 1 score ≥ 1 but NMSS Domain 5 score = 0 were excluded from the PD-SCD subgroup.

For the PD-SCD subgroup screening, NMSS Domain 5 includes three rater-administered items (Item 16: difficulty sustaining concentration during activities; Item 17: forgetting recently told information or events in the last few days; Item 18: forgetting to perform intended actions). Patients with UPDRS I Item 1 score ≥ 1 but NMSS Domain 5 score = 0 were excluded from the PD-SCD subgroup.

The MoCA score was further subdivided into four cognitive domain scores. Specifically, the “Visuospatial/Executive Function” domain includes the trail-making task, cube copying, and clock copying items; the “Memory” domain integrates delayed recall and orientation items; the “Attention” domain combines attention and language assessment items; and the “Language” domain incorporates language, naming, and abstraction items from the MoCA (Vogel et al., 2015; Mills et al., 2020).

### 2.3 MRI acquisition and processing

MRI data were acquired on a 3.0 T Siemens Prisma MRI system (Siemens, Germany) with a 20-channel head coil in the morning. PD patients underwent scanning in the medication-off state, following a 12-h withdrawal of antiparkinsonian medications. Earplugs and foam pads were used to reduce noise and head motion. A three-dimensional (3D) magnetization-prepared rapid acquisition gradient echo (MP-RAGE) sequence was used to obtain T1-weighted (T1w) anatomical images. The parameters of this sequence were as follows: repetition time (TR) = 2,300 ms, echo time (TE) = 2.98 ms, inversion time (TI) = 900 ms, flip angle (FA) = 9°, slice thickness = 1 mm, slices = 192, field of view (FOV) = 256 × 256 mm2, matrix size = 256 × 256, voxel size = 1 × 1 × 1 mm3, and acquisition time (TA) = 5 min 30 s.

Structural images were processed using FreeSurfer version 7.2.0. Automated segmentation of bilateral amygdala subregions was performed, and the left and right volumes of each subregion were summed to yield total volumetric measures for nine nuclei: lateral, basal, accessory basal, anterior amygdaloid area, central, medial, cortical, cortico-amygdaloid transition area, and paralaminar nuclei. Estimated total intracranial volume (eTIV) was calculated to account for interindividual differences in head size. All segmentations were visually inspected for quality assurance by two experienced radiologists. The visual inspection criteria primarily included: (1) no significant motion artifacts, signal inhomogeneity, or susceptibility artifacts affecting the amygdala region; and (2) accurate delineation of amygdala boundaries relative to adjacent structures (e.g., hippocampus, entorhinal cortex, and white matter). Regarding the handling of poor segmentations, to maintain methodological consistency and avoid rater bias, no manual edits or data reprocessing were performed. Two PD patients who failed to meet the visual inspection criteria were excluded from the final analysis.

### 2.4 Statistical analysis

Statistical analyses were conducted using SPSS version 26.0. Group differences in demographic, clinical, and imaging variables were assessed using analysis of variance, chi-square tests, or non-parametric tests as appropriate. Bonferroni correction was applied for multiple comparisons. Spearman correlation analyses examined associations between amygdala subregional volumes and cognitive measures.

To evaluate the independent association between amygdala subregional volumes and cognitive classification, multinomial logistic regression was conducted with PD-MCI as the reference category. The model adjusted for clinical covariates including age, sex, education, LEDD, disease duration, H-Y stage, UPDRS-III scores, and eTIV. Prior to modeling, all continuous variables were standardized to z-scores (mean = 0, SD = 1). Statistical significance was set at corrected *p* < 0.05.

## 3 Results

### 3.1 Clinical and cognitive characteristics

A total of 68 patients with PD and 27 HCs were included in the analysis, classified into PD-NC (*n* = 21), PD-SCD (*n* = 20), PD-MCI (*n* = 27), and HC (*n* = 27) groups.

The demographic and clinical variables of PD-NC, PD-SCD, PD-MCI and HC groups are summarized in Table 1. No significant differences were observed in gender, age, or educational level among the four groups after Bonferroni correction (all *p* > 0.05). Among PD subgroups, disease duration, H&Y stage, LEDD, and UPDRS II/III scores also showed no significant intergroup differences. Significant intergroup differences were noted in UPDRS I, Item 1 (*F* = 50.347, *p* < 0.001), with PD-MCI scoring higher than PD-SCD and PD-NC (*post hoc p* < 0.05). FAB scores approached significance (*F* = 2.959, *p* = 0.059), with PD-MCI showing a trend of lower scores. The MoCA total score and subdomains (visuospatial/executive, memory, attention and language) all exhibited significant differences (all p < 0.001), with PD-MCI having the lowest scores. For emotional assessments, HAM-D (*F* = 5.076, *p* = 0.003) and HAM-A (*F* = 8.437, *p* < 0.001) scores were significantly higher in all PD subgroups than in HC (post hoc *p* < 0.05), with no differences among PD subgroups.

**Table 1:** Intergroup comparisons of demographic features and clinical profiles.

	PD-MCI (*n* = 27)	PD-SCD (*n* = 20)	PD-NC (*n* = 21)	HC (*n* = 27)	Statistics
Gender (M/F)	15/12	13/7	13/8	12/15	Pearson’s Chi-Square = 2.416, *p* = 0.491
Age (years)	64.67 ± 6.58	66.75 ± 7.62	61.81 ± 11.40	62.96 ± 8.59	*F* = 1.318, *p* = 0.273
Education (years)	9.11 ± 2.97	10.65 ± 3.01	10.52 ± 2.68	9.63 ± 4.07	*F* = 1.189, *p* = 0.319
Duration (years)	4.87 ± 3.79	3.53 ± 2.30	4.14 ± 2.90	NA	*F* = 1.069, *p* = 0.349
H&Y	2.13 ± 0.78	1.88 ± 0.74	2.14 ± 0.67	NA	*F* = 0.883, *p* = 0.418
LEDD (mg)	460.65 ± 189.50	359.38 ± 223.01	368.45 ± 151.84	NA	*F* = 2.123, *p* = 0.128
UPDRS-I, Item 1	2.37 ± 1.08^ab^	1.35 ± 0.81^b^	0 ± 0	NA	*F* = 50.347, *p* < 0.001
UPDRS-II	10.48 ± 5.36	10.30 ± 4.41	9.10 ± 3.75	NA	*F* = 0.587, *p* = 0.559
UPDRS-III	24.19 ± 12.50	19.90 ± 10.06	22.90 ± 10.08	NA	*F* = 0.872, *p* = 0.423
FAB	16.59 ± 1.60^c^	17.10 ± 1.67	17.48 ± 0.75	NA	F = 2.959, *p* = 0.059
MoCA	22.56 ± 1.85^abc^	26.85 ± 1.27^c^	27.81 ± 1.40	28.59 ± 1.22	*F* = 94.741, *p* < 0.001
MoCA-visuospatial/executive	3.67 ± 1.14^abc^	4.45 ± 0.83	4.57 ± 0.60	4.78 ± 0.42	*F* = 9.693, *p* < 0.001
MoCA-memory	6.85 ± 1.10^abc^	8.95 ± 1.19^bc^	9.67 ± 1.15	9.96 ± 0.98	*F* = 42.806, *p* < 0.001
MoCA-attention	7.74 ± 1.20^abc^	8.85 ± 0.37	8.86 ± 0.36	8.96 ± 0.19	*F* = 18.252, *p* < 0.001
MoCA-language	6.37 ± 1.28^abc^	7.15 ± 0.93^c^	7.29 ± 0.72^c^	7.85 ± 0.36	*F* = 12.583, *p* < 0.001
HAM-D	6.81 ± 5.41^c^	7.55 ± 5.55^c^	5.33 ± 4.67	2.89 ± 1.99	F = 5.076, *p* = 0.003
HAM-A	10.93 ± 8.79^c^	9.30 ± 5.76^c^	8.71 ± 7.45^c^	2.56 ± 1.87	F = 8.437, *p* < 0.001

PD, Parkinson’s disease; MCI, mild cognitive impairment; SCD, subjective cognitive decline; NC, normal cognition; HC, healthy control; H&Y, Hoehn & Yahr scale; LEDD, levodopa equivalent daily dose; UPDRS, Unified Parkinson’s Disease Rating Scale; FAB, Frontal Assessment Battery; MoCA, Montreal Cognitive Assessment; HAM-D, 17-item Hamilton Depression Rating Scale; HAM-A, Hamilton Anxiety Rating Scale; NA, not applicable. Post hoc two-sample *t*-test: ^a^ significant difference compared to the PD-SCD group; ^b^ significant difference compared to the PD-NC group; ^c^ significant difference compared to the HC group. Significance levels: * *p* < 0.05, ** *p* < 0.01, *** *p* < 0.001, Bonferroni-corrected.

### 3.2 Amygdala subregional volumes

**Figure 1:**
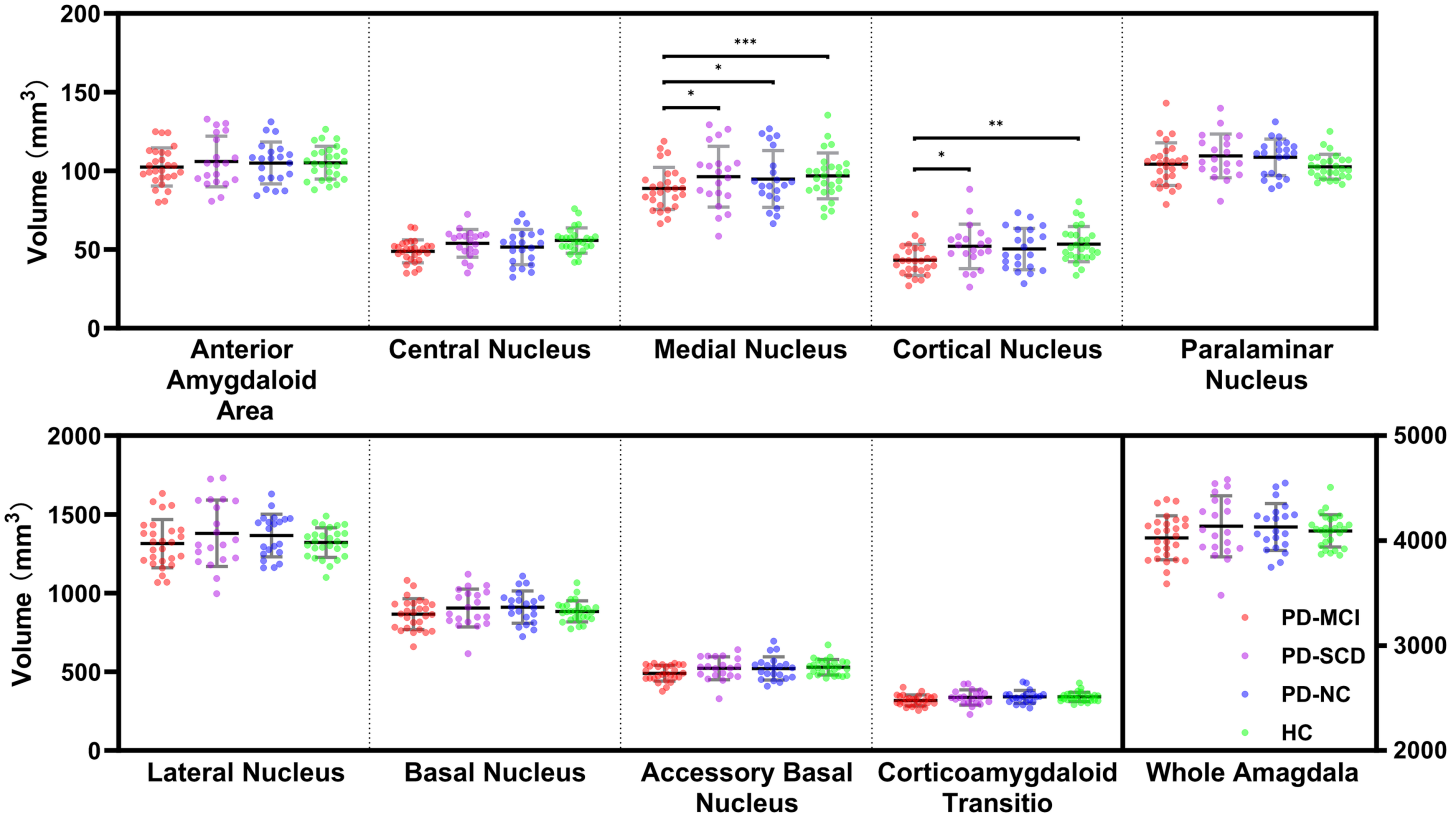
Intergroup differences in amygdala subregional volumes among PD patients across cognitive stages and HCs. Comparison of the volumes of amygdala subregions among PD-MCI (*N* = 27), PD-SCD (*N* = 20), PD-NC (*N* = 21) and HC (*N* = 27) with ANOVA analysis. The scatter dot plots show the volumes of nine specific amygdala subnuclei and the whole amygdala. The horizontal lines represent the mean and standard deviation (SD). Error bars represent the standard error of the mean. Asterisks indicate statistically significant group differences (*: *p* < 0.05, **: *p* < 0.01,***: *p* < 0.001). PD, Parkinson’s disease; MCI, mild cognitive impairment; SCD, subjective cognitive decline; NC, non cognitive impairment; HC, healthy control. Different colors represent different subgroups: PD-NC (blue), PD-SCD (purple), PD-MCI (red) and HC (green).

Volumetric analysis of amygdala subregions revealed significant differences among the four groups (PD-MCI, PD-SCD, PD-NC, and HC). As detailed in Table 2 and illustrated in Figure 1, one-way ANOVA showed significant group effects for the medial nucleus (*F* = 3.701, *p* = 0.015) and the cortical nucleus (*F* = 3.043, *p* = 0.033). Post-hoc comparisons indicated that the PD-MCI group exhibited significantly smaller volumes in the medial nucleus compared to the PD-SCD, PD-NC, and HC groups. Similarly, the volume of the cortical nucleus in the PD-MCI group was significantly reduced relative to the other three groups. The volumetric profiles of these and other subregions across groups are visually presented in Figure 1. No significant volumetric differences were found in the lateral nucleus, basal nucleus, accessory basal nucleus, anterior amygdala area, central nucleus, cortico-amygdaloid transition area, or paralaminar nucleus among the groups (all *p* > 0.05). Furthermore, the whole amygdala volume did not differ significantly across groups (*F* = 1.299, *p* = 0.280). eTIV did not differ significantly among groups (*F* = 2.311, *p* = 0.081), and its inclusion as a covariate did not alter the significance of the aforementioned subregional findings.

**Table 2:** Volumetric analysis of amygdala subnuclei across different cognitive stages in patients with PD and HCs.

	PD-MCI (*n* = 27)	PD-SCD (*n* = 20)	PD-NC (*n* = 21)	HC (*n* = 27)	Statistics
Lateral nucleus	1314.56 ± 152.96	1380.01 ± 210.47	1365.84 ± 135.78	1320.93 ± 94.49	*F* = 1.082, *p* = 0.361
Basal nucleus	865.77 ± 98.14	905.24 ± 120.14	910.97 ± 102.20	883.98 ± 66.41	*F* = 1.093, *p* = 0.356
Accessory basal nucleus	491.11 ± 49.83	522.47 ± 72.44	521.16 ± 73.41	529.06 ± 49.25	*F* = 2.051, *p* = 0.112
Anterior amygdala area	102.43 ± 12.16	105.90 ± 16.11	104.98 ± 13.29	105.16 ± 10.43	*F* = 0.344, *p* = 0.794
Central nucleus	88.84 ± 13.37	96.23 ± 19.27	94.82 ± 18.02	96.75 ± 14.55	*F* = 1.329, *p* = 0.270
Medial nucleus	43.30 ± 9.91^abc^	52.03 ± 14.18	50.30 ± 13.13	53.42 ± 11.14	*F* = 3.701, *p* = 0.015
Cortical nucleus	48.84 ± 7.22^ac^	53.95 ± 8.87	51.63 ± 11.17	55.75 ± 8.04	*F* = 3.043, *p* = 0.033
Cortico-amygdaloid transition area	318.36 ± 34.31	337.14 ± 47.58	340.91 ± 40.97	340.39 ± 30.13	*F* = 1.318, *p* = 0.273
Paralaminar nucleus	104.19 ± 13.51	109.42 ± 13.90	108.57 ± 11.57	102.61 ± 7.94	*F* = 2.061, *p* = 0.111
Whole amygdala area	3377.40 ± 349.24	3562.38 ± 484.67	3549.18 ± 372.10	3488.05 ± 256.74	*F* = 1.299, *p* = 0.280
eTIV (10^4^)	145.00 ± 16.12	155.40 ± 19.43	148.29 ± 14.84	143.44 ± 15.52	*F* = 2.311, *p* = 0.081

PD, Parkinson’s disease; MCI, mild cognitive impairment; SCD, subjective cognitive decline; NC, normal cognition; HC, healthy control; eTIV, estimated total intracranial volume. Post hoc two-sample *t*-test: ^a^ significant difference compared to the PD-SCD group; ^b^ significant difference compared to the PD-NC group; ^c^ significant difference compared to the HC group. Significance levels: * *p* < 0.05, ** *p* < 0.01, *** *p* < 0.001.

### 3.3 Associations with cognitive performance

**Figure 2:**
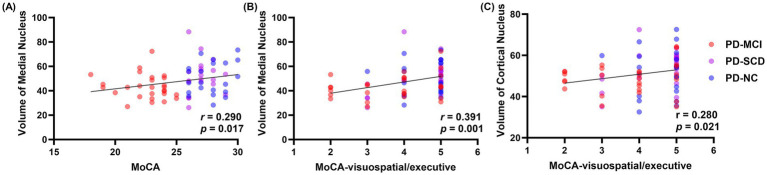
Correlations between amygdala subregional volumes and cognitive scores in Parkinson’s disease patients. Scatter plots showing the correlation analysis between subnuclei volumes and MoCA scores. **(A)** Correlation between the volume of the medial nucleus and total MoCA scores. **(B)** Correlation between the volume of the medial nucleus and MoCA visuospatial/executive sub-scores. **(C)** Correlation between the volume of the cortical nucleus and MoCA visuospatial/executive sub-scores. The solid lines represent the linear regression fit. *r* represents the correlation coefficient; *p* indicates the significance level. Different colors represent different subgroups: PD-NC (blue), PD-SCD (purple), and PD-MCI (red).

The volume of the medial nucleus, which was significantly atrophied in the PD-MCI group, was significantly positively correlated with MoCA scores (*r* = 0.290, *p* = 0.017), and with the visuospatial/executive function performance of detailed neuropsychological tests (*r* = 0.391, *p* = 0.001). No correlations were found between medial nucleus volume and the memory, attention and language subcategories of MoCA (all *p* > 0.05). Similarly, the volume of the cortical nucleus was also positively correlated with the MoCA visuospatial/executive score (*r* = 0.280, *p* = 0.021). As demonstrated in Figure 2.

In contrast, the volumes of the total amygdala, lateral nucleus, basal nucleus, central nucleus, and other subregions were not significantly correlated with MoCA total scores or its subscores (all *p* > 0.05).

### 3.4 Multinomial logistic regression analysis

Multinomial logistic regression analysis revealed an association between medial amygdala (MeA) volume and cognitive subgroups in PD patients. The model was overall significant (χ^2^ = 6.986, *p* = 0.03), and MeA volume was significantly associated with cognitive subgroup classification (*p* = 0.03). Compared to PD-MCI, per standard deviation increase in MeA volume was associated with marginally significantly increased odds of PD-NC (OR = 1.053, 95% CI: 1.000–1.110; B = 0.052, *p* = 0.050) and significantly increased odds of PD-SCD (OR = 1.064, 95% CI: 1.009–1.122; B = 0.062, *p* = 0.021). These findings indicate that larger MeA volume correlates with a higher likelihood of belonging to cognitively preserved subgroups in PD, suggesting a potential cognitive protective role of MeA volume.

## 4 Discussion

This study identified selective atrophy of the medial and cortical amygdala subregions as a key structural correlate of PD-MCI, while PD-SCD patients showed no detectable changes in these subregions—findings that deepen our understanding of the neural progression of PD-related cognitive impairment. Notably, this observation aligns with the increasingly recognized “cognitive continuum” of PD, which posits a gradual transition from preserved cognition to overt dementia (Schumacher et al., 2023; Kang et al., 2024), and provides novel structural evidence for stratifying the early stages of this continuum. Prior studies have primarily focused on cortical or hippocampal alterations in PD cognitive impairment (Silva-Rodríguez et al., 2025), but our findings highlight subcortical limbic subregions as critical nodes in the pathogenesis of PD-related cognitive decline, filling the gap in understanding subregional vulnerability within the amygdala across different cognitive stages. This subregional vulnerability aligns with neuroimaging research demonstrating that PD-MCI is characterized by targeted damage to associative brain regions rather than widespread neurodegeneration, and supports the dual syndrome hypothesis that distinguishes frontal-striatal from posterior cortical cognitive impairments in PD (Pourzinal et al., 2022).

The cortical amygdala’s involvement in PD-MCI further reinforces the role of associative processing deficits in early cognitive decline, as this subregion integrates sensory and cognitive information to support higher-order thinking (Zhang et al., 2023). Emerging neuropathological evidence indicates that *α*-synuclein aggregates within the amygdala may spread to interconnected prefrontal cortical regions via transsynaptic pathways (Zhou et al., 2024; Fujita et al., 2023), thereby disrupting the prefrontal-limbic network that mediates goal-directed behavior, decision-making, and emotional regulation — all of which constitute the core functional domains impaired in PD-MCI (Devignes et al., 2022). Its atrophy may contribute to the visuospatial and executive dysfunction typical of PD-MCI, mirroring findings that amygdala subregional damage correlates with specific cognitive domain impairments in PD (Schröter et al., 2025; Villar-Conde et al., 2023). Notably, whole-amygdala volume failed to differentiate between PD subgroups, emphasizing the necessity of subregional analysis— a point underscored by structural connectome studies showing that network-specific alterations, rather than global volume changes, distinguish PD-MCI from cognitively preserved PD. However, we acknowledge that the absence of a significant whole-amygdala volume difference may also reflect limited statistical power due to our relatively modest sample size, which could have prevented detection of subtle global volumetric changes. This finding is particularly meaningful because it highlights the limitations of gross structural assessments in capturing early, focal neurodegeneration, which may be masked by preserved volume in other amygdala subregions (e.g., lateral or basal nuclei) that are less vulnerable to early PD pathology (Laansma et al., 2024; Wang et al., 2023). This specificity highlights the limitations of gross structural assessments in capturing early cognitive vulnerability, as supported by other MRI research indicating that microstructural and connectivity measures may be more sensitive to early PD-related changes (Wang et al., 2023; Huang et al., 2023; Zhang et al., 2025). The absence of amygdala subregional atrophy in PD-SCD offers critical insights into the early stages of cognitive complaint in PD, aligning with systematic reviews that frame PD-SCD as a heterogeneous entity with functional rather than structural correlates (Pourzinal et al., 2022). PD-SCD patients are at heightened risk of progressing to PD-MCI, but their preserved amygdala structure suggests a window for early interventions targeting functional connectivity or non-motor symptoms like anxiety and depression—conditions closely linked to amygdala circuit dysfunction in PD (Qu et al., 2024).

Our finding that medial amygdala volume is independently associated with cognitive resilience in PD supports its potential as a structural biomarker, joining a growing list of neuroanatomical correlates of cognitive function in PD (Alzahrani and Venneri, 2015). Cognitive reserve—defined as the ability to maintain cognitive function despite underlying neurodegenerative pathology (Yang et al., 2024; Huang et al., 2025)—has emerged as a key target in PD research (Le Stanc et al., 2024), and the present study suggests that the medial amygdala may serve as a neural substrate mediating this reserve capacity. Patients with larger medial amygdala volumes may retain sufficient prefrontal-limbic connectivity to compensate for early pathological changes in PD, suggesting that structural integrity of the medial amygdala may serve as a neural substrate of cognitive reserve, potentially buffering against the transition from subjective decline to objective impairment. This is consistent with previous findings that in PD patients comorbid with small vessel disease (SVD), volumetric atrophy patterns of amygdala subregions act as an independent risk factor for declines in cognitive control and processing abilities and are associated with subsequent cognitive decline (Cheng et al., 2024). This marker’s prognostic value complements other imaging-based biomarkers, such as hippocampal subregional volumes and cortical thickness, which have been linked to memory and executive deficits in PD (Ryman and Poston, 2019). When combined with body fluid biomarkers like serum NfL and CSF *α*-synuclein, amygdala subregional volumetry can improve prognostic accuracy for PD cognitive progression—consistent with multi-modal biomarker studies showing enhanced prognostic power when integrating structural and molecular measures (Goldman and Sieg, 2020; Leaver and Poston, 2015). While our findings highlight the sensitivity of subregional amygdala measures, it is important to note that structural abnormalities in cortical regions may emerge earlier than suggested by traditional α-synuclein propagation models, as recently demonstrated in the pre-decline stages of PD (Seo et al., 2025). Because our study focused specifically on the amygdala, we did not perform comparative analyses against multi-regional markers (e.g., global cortical thickness or hippocampal subfields). Therefore, future longitudinal studies incorporating multi-regional comparisons are needed to determine whether amygdala subregional atrophy offers independent, superior, or complementary predictive value for early cognitive decline.

Beyond the need for such multi-regional investigations, several methodological limitations of the present study should also be acknowledged. Chiefly, the cross-sectional design prevents establishing causal relationships between amygdala subregional atrophy and cognitive decline. This is compounded by our single-center sample, which may limit generalizability, although larger multi-cohort studies have replicated subregional limbic involvement in PD cognitive impairment. Beyond these study design constraints, our analytical approach utilized summed bilateral volumes rather than performing hemispheric laterality analyses. Given that PD pathology is characteristically lateralized at onset and cognitive symptoms may correlate with asymmetric neurodegeneration, bilateral summation of amygdala subregional volumes could therefore mask unilateral atrophy patterns and prevent detection of hemisphere-specific structure-cognition associations, thereby limiting our ability to interpret hemisphere-specific pathophysiological mechanisms. Consequently, subsequent research should systematically explore lateralized amygdala alterations and their associations with asymmetric motor and cognitive symptoms.

In terms of clinical evaluation, our classification of PD-MCI relied primarily on the MoCA, representing a Level I screening classification per MDS guidelines. Because this approach may not fully align with Level II classifications based on comprehensive neuropsychological testing, implementing multi-domain assessments in the future would allow for finer-grained analyses of subregion-cognitive domain relationships, particularly since PD cognitive impairment is heterogeneous and may map to distinct neural substrates. Furthermore, future studies should also integrate tau PET imaging to clarify the interaction between *α*-synuclein and tau pathologies in amygdala subregional degeneration.

In conclusion, our results demonstrate that selective medial and cortical amygdala atrophy characterizes PD-MCI but not PD-SCD, providing a structural basis for cognitive staging in PD. These findings build on prior work linking amygdala subregional dysfunction to α-synuclein propagation and cognitive decline, and emphasize the clinical value of targeted subregional analysis over gross structural measures. As research advances toward multi-modal biomarker panels and personalized interventions, amygdala subregional volumetry will likely play an increasingly important role in early risk stratification, prognosis, and treatment monitoring for PD-related cognitive impairment.

## Data Availability

The raw data supporting the conclusions of this article will be made available by the authors, without undue reservation.

## References

[ref1] Alzahrani H. Venneri A. (2015). Cognitive and neuroanatomical correlates of neuropsychiatric symptoms in Parkinson's disease: a systematic review. J. Neurol. Sci. 356, 32–44. doi: 10.1016/j.jns.2015.06.037, 26123201

[ref2] Ay U. Yıldırım Z. Erdogdu E. Kiçik A. Ozturk-Isik E. Demiralp T. . (2022). Shrinkage of olfactory amygdala connotes cognitive impairment in patients with Parkinson's disease. Cogn. Neurodyn. 17, 1309–1320. doi: 10.1007/s11571-022-09887-y, 37786655 PMC10542039

[ref3] Ben-Zion Z. Korem N. Spiller T. R. Duek O. Keynan J. N. Admon R. . (2022). Longitudinal volumetric evaluation of hippocampus and amygdala subregions in recent trauma survivors. Mol. Psychiatry 28, 657–667. doi: 10.1038/s41380-022-01842-x, 36280750 PMC9918676

[ref4] Braak H. de Vos R. A. Jansen E. N. Bratzke H. Braak E. (1998). Neuropathological hallmarks of Alzheimer's and Parkinson's diseases. Prog. Brain Res. 117, 267–285. doi: 10.1016/S0079-6123(08)64021-2 9932414

[ref5] Chen J. Liang C. Wang F. Zhu Y. Zhu L. Chen J. . (2026). Potential biofluid markers for cognitive impairment in Parkinson's disease. Neural Regen. Res. 21, 281–295. doi: 10.4103/NRR.NRR-D-24-00592, 39851136 PMC12094573

[ref6] Cheng Z. Yang L. Li J. Chen Y. Liang P. Wang Y. . (2024). Cognitive impairment and amygdala subregion volumes in elderly with cerebral small vessel disease: a large prospective cohort study. Neurobiol. Dis. 202:106716. doi: 10.1016/j.nbd.2024.106716, 39490683

[ref7] Devignes Q. Bordier C. Viard R. Defebvre L. Kuchcinski G. Leentjens A. F. G. . (2022). Resting-state functional connectivity in Frontostriatal and posterior cortical subtypes in Parkinson's disease-mild cognitive impairment. Mov. Disord. 37, 502–512. doi: 10.1002/mds.28888, 34918782

[ref8] Flores-Cuadrado A. Ubeda-Bañon I. Saiz-Sanchez D. de la Rosa-Prieto C. Martinez-Marcos A. (2014). Α-Synuclein staging in the amygdala of a Parkinson's disease model: cell types involved. Eur. J. Neurosci. 41, 137–146. doi: 10.1111/ejn.12763, 25345880

[ref9] Flores-Torres M. H. Bjornevik K. Hung A. Y. Healy B. C. Schwarzschild M. A. Blacker D. . (2023). Subjective cognitive decline in women with features suggestive of prodromal Parkinson's disease. Mov. Disord. 38, 1473–1482. doi: 10.1002/mds.29503, 37315105 PMC10524634

[ref10] Fujita K. Homma H. Jin M. Yoshioka Y. Jin X. Saito Y. . (2023). Mutant α-synuclein propagates via the lymphatic system of the brain in the monomeric state. Cell Rep. 42:112962. doi: 10.1016/j.celrep.2023.112962, 37591248

[ref11] Goldman J. G. Sieg E. (2020). Cognitive impairment and dementia in Parkinson disease. Clin. Geriatr. Med. 36, 365–377. doi: 10.1016/j.cger.2020.01.001, 32222308

[ref12] Huang X. He Q. Ruan X. Li Y. Kuang Z. Wang M. . (2023). Structural connectivity from DTI to predict mild cognitive impairment in de novo Parkinson's disease. Neuroimage Clin. 41:103548. doi: 10.1016/j.nicl.2023.103548, 38061176 PMC10755095

[ref13] Huang X. Ling Y. Tan S. Bai Z. Shen S. Wang H. . (2025). Cognitive reserve, frailty status, and risk of neurodegenerative diseases: a prospective cohort study. NPJ Parkinsons Dis. 12:20. doi: 10.1038/s41531-025-01231-5, 41372216 PMC12804963

[ref14] Kang M. Li C. Mahajan A. Spat-Lemus J. Durape S. Chen J. . (2024). Subjective cognitive decline plus and longitudinal assessment and risk for cognitive impairment. JAMA Psychiatry 81, 993–1002. doi: 10.1001/jamapsychiatry.2024.1678, 38959008 PMC11223054

[ref15] Kiene F. Hildebrandt H. Roheger M. (2025). Will I get dementia? – symptoms and worries of individuals affected by subjective cognitive decline. Alzheimers Dement. 20:20(S3). doi: 10.1002/alz.086835, 41531421

[ref16] Laansma M. A. Zhao Y. van Heese E. M. Bright J. K. Owens-Walton C. Al-Bachari S. . (2024). A worldwide study of subcortical shape as a marker for clinical staging in Parkinson's disease. NPJ Parkinsons Dis. 10:223. doi: 10.1038/s41531-024-00825-9, 39557903 PMC11574005

[ref17] Lai T. T. Xiang W. Stanojlovic M. Käufer C. Feja M. Lau K. . (2025). The basolateral amygdala and striatum propagate alpha-synuclein pathology causing increased fear response in a Parkinson's disease model. Brain Behav. Immun. 128, 469–486. doi: 10.1016/j.bbi.2025.04.025, 40274000

[ref18] Le Stanc L. Lunven M. Giavazzi M. Sliwinski A. Youssov K. Bachoud-Lévi A. C. . (2024). Cognitive reserve involves decision making and is associated with left parietal and hippocampal hypertrophy in neurodegeneration. Commun. Biol. 7:741. doi: 10.1038/s42003-024-06416-x, 38890487 PMC11189446

[ref19] Leaver K. Poston K. L. (2015). Do CSF biomarkers predict progression to cognitive impairment in Parkinson's disease patients? A systematic review. Neuropsychol. Rev. 25, 411–423. doi: 10.1007/s11065-015-9307-8, 26626621 PMC5152566

[ref20] Leaver A. M. Vasavada M. Kubicki A. Wade B. Loureiro J. Hellemann G. . (2020). Hippocampal subregions and networks linked with antidepressant response to electroconvulsive therapy. Mol. Psychiatry 26, 4288–4299. doi: 10.1038/s41380-020-0666-z, 32029885 PMC7415508

[ref21] Lee J. E. Cho K. H. Kim M. Sohn Y. H. Lee P. H. (2011). The pattern of cortical atrophy in Parkinson's disease with mild cognitive impairment according to the timing of cognitive dysfunction. J. Neurol. 259, 469–473. doi: 10.1007/s00415-011-6203-x, 21818688

[ref22] Leite Silva A. B. R. Gonçalves de Oliveira R. W. Diógenes G. P. de Castro Aguiar M. F. Sallem C. C. Lima M. P. P. . (2022). Premotor, nonmotor and motor symptoms of Parkinson's disease: a new clinical state of the art. Ageing Res. Rev. 84:101834. doi: 10.1016/j.arr.2022.101834, 36581178

[ref23] Loo R. T. J. Pavelka L. Mangone G. Khoury F. Vidailhet M. Corvol J.-C. . (2025). Multi-cohort machine learning identifies tictors of cognitive impairment in Parkinson's disease. NPJ Digit. Med. 8:482. doi: 10.1038/s41746-025-01862-1, 40715642 PMC12297691

[ref24] Mills K. A. Schneider R. B. Saint-Hilaire M. Ross G. W. Hauser R. A. Lang A. E. . (2020). Cognitive impairment in Parkinson's disease: associations between subjective and objective cognitive decline in a large longitudinal study. Parkinsonism Relat. Disord. 80, 127–132. doi: 10.1016/j.parkreldis.2020.09.028, 32987359 PMC12396391

[ref25] Oedekoven C. Egeri L. Jessen F. Wagner M. Dodel R. (2021). Subjective cognitive decline in idiopathic Parkinson’s disease: a systematic review. Ageing Res. Rev. 74:101508. doi: 10.1016/j.arr.2021.101508, 34740867

[ref26] Oh Y. Kim J.-S. Lyoo C. H. Park G. Kim H. (2024). Spatiotemporal progression patterns of dopamine availability and deep Gray matter volume in Parkinson disease-related cognitive impairment. Neurology 103:e209498. doi: 10.1212/WNL.0000000000209498, 38885485

[ref27] Oikonomou P. Akhoundi F. H. Olfati N. Litvan I. (2025). Characteristics and mechanisms of cognitive impairment in Parkinson disease. Nat. Rev. Neurol. 22, 90–109. doi: 10.1038/s41582-025-01163-x, 41339536

[ref28] Oltra J. Uribe C. Segura B. Campabadal A. Inguanzo A. Monté-Rubio G. C. . (2022). Brain atrophy pattern in de novo Parkinson's disease with probable RBD associated with cognitive impairment. NPJ Parkinsons Dis. 8:60. doi: 10.1038/s41531-022-00326-7, 35610256 PMC9130201

[ref29] Park G. Ha J. Lee J. S. Ahn J. H. Cho J. W. Seo S. W. . (2025). Data-driven, cross-sectional image-based subtyping and staging of brain volumetric changes in Parkinson's disease. Neurobiol. Dis. 212:106970. doi: 10.1016/j.nbd.2025.106970, 40418995

[ref30] Postuma R. B. Berg D. Stern M. Poewe W. Olanow C. W. Oertel W. . (2015). MDS clinical diagnostic criteria for Parkinson's disease. Mov. Disord. 30, 1591–1601. doi: 10.1002/mds.26424, 26474316

[ref31] Pourzinal D. Yang J. Lawson R. A. McMahon K. L. Byrne G. J. Dissanayaka N. N. (2022). Systematic review of data-driven cognitive subtypes in Parkinson disease. Eur. J. Neurol. 29, 3395–3417. doi: 10.1111/ene.15481, 35781745 PMC9796227

[ref32] Qu M. Gao B. Jiang Y. Li Y. Pei C. Xie L. . (2024). Atrophy patterns in hippocampus and amygdala subregions of depressed patients with Parkinson's disease. Brain Imaging Behav. 18, 475–484. doi: 10.1007/s11682-023-00844-9, 38170304 PMC11222218

[ref33] Ryman S. G. Poston K. L. (2019). MRI biomarkers of motor and non-motor symptoms in Parkinson's disease. Parkinsonism Relat. Disord. 73, 85–93. doi: 10.1016/j.parkreldis.2019.10.002, 31629653 PMC7145760

[ref34] Schapira A. H. V. Chaudhuri K. R. Jenner P. (2017). Non-motor features of Parkinson disease. Nat. Rev. Neurosci. 18, 435–450. doi: 10.1038/nrn.2017.62 28592904

[ref35] Schröter N. Matinpalo L. Hosp J. A. Reisert M. Philipsen L. Jost W. H. . (2025). Amygdala neurodegeneration differentiates brain-first and body-first Parkinson's disease: an MRI study. Parkinsonism Relat. Disord. 135:107827. doi: 10.1016/j.parkreldis.2025.107827, 40209563

[ref36] Schumacher J. Kanel P. Dyrba M. Storch A. Bohnen N. I. Teipel S. . (2023). Structural and molecular cholinergic imaging markers of cognitive decline in Parkinson's disease. Brain 146, 4964–4973. doi: 10.1093/brain/awad226, 37403733 PMC10689921

[ref37] Seo K. Oyama G. Yamamoto T. (2025). Subregional analysis of the amygdala, thalamus, and hypothalamus at the pre-decline stage in Parkinson's disease with later cognitive impairment. Front. Aging Neurosci. 17:1588027. doi: 10.3389/fnagi.2025.1588027, 40416735 PMC12098468

[ref38] Silva-Rodríguez J. Labrador-Espinosa M. Á. Castro-Labrador S. Muñoz-Delgado L. Franco-Rosado P. Castellano-Guerrero A. M. . (2025). Imaging biomarkers of cortical neurodegeneration underlying cognitive impairment in Parkinson's disease. Eur. J. Nucl. Med. Mol. Imaging 52, 2002–2014. doi: 10.1007/s00259-025-07070-z, 39888421 PMC12014801

[ref39] Sivaranjini S. Sujatha C. M. (2021). Morphological analysis of subcortical structures for assessment of cognitive dysfunction in Parkinson's disease using multi-atlas based segmentation. Cogn. Neurodyn. 15, 835–845. doi: 10.1007/s11571-021-09671-4, 34603545 PMC8448821

[ref40] Svenningsson P. Westman E. Ballard C. Aarsland D. (2012). Cognitive impairment in patients with Parkinson's disease: diagnosis, biomarkers, and treatment. Lancet Neurol. 11, 697–707. doi: 10.1016/S1474-4422(12)70152-7, 22814541

[ref41] Torres E. R. S. Stanojlovic M. Zelikowsky M. Bonsberger J. Hean S. Mulligan C. . (2021). Alpha-synuclein pathology, microgliosis, and parvalbumin neuron loss in the amygdala associated with enhanced fear in the Thy1-aSyn model of Parkinson's disease. Neurobiol. Dis. 158:105478. doi: 10.1016/j.nbd.2021.105478, 34390837 PMC8447919

[ref42] Van Den Berge N. Ferreira N. Mikkelsen T. W. Alstrup A. K. O. Tamgüney G. Karlsson P. . (2021). Ageing promotes pathological alpha-synuclein propagation and autonomic dysfunction in wild-type rats. Brain 144, 1853–1868. doi: 10.1093/brain/awab061, 33880502 PMC8320301

[ref43] Villar-Conde S. Astillero-Lopez V. Gonzalez-Rodriguez M. Saiz-Sanchez D. Martinez-Marcos A. Ubeda-Banon I. . (2023). Synaptic involvement of the human amygdala in Parkinson's disease. Mol. Cell. Proteomics 22:100673. doi: 10.1016/j.mcpro.2023.100673, 37947401 PMC10700869

[ref44] Vogel S. J. Banks S. J. Cummings J. L. Miller J. B. (2015). Concordance of the Montreal cognitive assessment with standard neuropsychological measures. Alzheimers Dement. 1, 289–294. doi: 10.1016/j.dadm.2015.05.002, 27239512 PMC4877929

[ref45] Wang J. Sun L. Chen L. Sun J. Xie Y. Tian D. . (2023). Common and distinct roles of amygdala subregional functional connectivity in non-motor symptoms of Parkinson's disease. NPJ Parkinsons Dis. 9:28. doi: 10.1038/s41531-023-00469-1, 36806219 PMC9938150

[ref46] Weintraub D. Marras C. Amara A. Anderson K. E. Chahine L. M. Eberly S. . (2024). Association between subjective cognitive complaints and incident functional impairment in Parkinson's disease. Mov. Disord. 39, 706–714. doi: 10.1002/mds.29725, 38318953

[ref47] Wen C. Hu H. Ou Y. N. Bi Y. L. Ma Y. H. Tan L. . (2021). Risk factors for subjective cognitive decline: the CABLE study. Transl. Psychiatry 11:576. doi: 10.1038/s41398-021-01711-1, 34753917 PMC8578345

[ref48] Yang W. Wang J. Dove A. Dunk M. M. Qi X. Bennett D. A. . (2024). Association of cognitive reserve with the risk of dementia in the UK biobank: role of polygenic factors. Br. J. Psychiatry 224, 213–220. doi: 10.1192/bjp.2024.13, 38328972

[ref49] Zhang Y. Li S. Yu J. Li R. Liao W. Chen Q. . (2025). Amygdala-centered fusional connections characterized nonmotor symptoms in Parkinson's disease. Cereb. Cortex 35:bhaf002. doi: 10.1093/cercor/bhaf002 39838822

[ref50] Zhang L. Zhang P. Dong Q. Zhao Z. Zheng W. Zhang J. . (2023). Fine-grained features characterize hippocampal and amygdaloid change pattern in Parkinson's disease and discriminate cognitive-deficit subtype. CNS Neurosci. Ther. 30:e14480. doi: 10.1111/cns.14480, 37849445 PMC10805398

[ref51] Zhou W. Daniels S. Singh V. Menard M. Escobar Galvis M. L. Chu H. Y. (2024). α-Synuclein aggregation decreases cortico-amygdala connectivity and impairs social behavior in mice. Neurobiol. Dis. 202:106702. doi: 10.1016/j.nbd.2024.106702, 39406290

[ref52] Zhu Y. Yang B. Zhou C. Gao C. Hu Y. Yin W. F. . (2022). Cortical atrophy is associated with cognitive impairment in Parkinson's disease: a combined analysis of cortical thickness and functional connectivity. Brain Imaging Behav. 16, 2586–2600. doi: 10.1007/s11682-022-00714-w, 36044168

